# Electrically Doped PNPN Tunnel Field-Effect Transistor Using Dual-Material Polarity Gate with Improved DC and Analog/RF Performance

**DOI:** 10.3390/mi14122149

**Published:** 2023-11-24

**Authors:** Chan Shan, Ying Liu, Yuan Wang, Rongsheng Cai, Lehui Su

**Affiliations:** 1 College of Ocean Information Engineering, Jimei University, Xiamen 361021, China; shanchan@jmu.edu.cn; 2 Department of Economics and Business, University of Navarra, 31006 Pamplona, Spain; 3 Department of Software Technology, Xiamen Institute of Software Technology, Xiamen 361021, China; liuyule0504@foxmail.com; 4 Faculty of Data Science, City University of Macau, Macau 999078, China; rongcheng1986@163.com; 5 College of Software, Quanzhou University of Information Engineering, Quanzhou 362000, China; rjxy@qziedu.cn

**Keywords:** tunnel FETs (TFETs), electrically doped, dual-material gate (DMG), band-to-band tunneling (BTBT), analog/RF performance

## Abstract

A new structure for PNPN tunnel field-effect transistors (TFETs) has been designed and simulated in this work. The proposed structure incorporates the polarity bias concept and the gate work function engineering to improve the DC and analog/RF figures of merit. The proposed device consists of a control gate (CG) and a polarity gate (PG), where the PG uses a dual-material gate (DMG) structure and is biased at −0.7 V to induce a P^+^ region in the source. The PNPN structure introduces a local minimum on the conduction band edge curve at the tunneling junction, which dramatically reduces the tunneling width. Furthermore, we show that incorporating the DMG architecture further enhances the drive current and improves the subthreshold slope (SS) characteristics by introducing an additional electric field peak. The numerical simulation reveals that the electrically doped PNPN TFET using DMG improves the DC and analog/RF performances in comparison to a conventional single-material gate (SMG) device.

## 1. Introduction

It has become increasingly difficult for the industry to continuously scale traditional Complementary Metal Oxide Semiconductor (CMOS) devices at the nanoscale level. At room temperature, the 60 mV/dec subthreshold slope acts as a limit on transistor scaling in Metal Oxide Semiconductor Field-Effect Transistors (MOSFETs). One such candidate is the Tunnel Field-Effect Transistor (TFET), whose subthreshold slope value at room temperature is less than 60 mV/decade [1]. In addition to these advantages, TFETs have two major disadvantages, namely low on-state current (I_ON_) and ambipolarity during switching [2]. In order to overcome I_ON_ and the ambipolar issue, we have recently proposed an in-built N^+^ pocket electrically doped TFET (ED-TFET) with and without an electrically doped drain, using the concept of polarity bias [3,4]. An in-built N^+^ pocket ED-TFET structure is very similar to a PNPN TFET structure, except that it does not require additional chemical doping for the narrow N^+^ pocket [5,6]. By applying a bias voltage at both the polarity gate and control gate, the principle of the polarity bias concept induces charge carriers that modulate tunneling barriers [7,8]. Consequently, no additional doping processes are required to build a narrow N^+^ pocket, thereby simplifying the manufacturing process [9].

In previous works, simulation studies have demonstrated that by replacing the single-material control gate structure in the double-gate TFET with a dual-material control gate structure, both the ON-current and subthreshold slope (SS) characteristics of the TFETs could be significantly improved [10,11]. For example, a new structure of Schottky tunneling MOSFET has been designed and simulated using floating gates and dual-material main gates to counter short-channel effects and improve analog/RF performances [12]. In addition, a dual-material control gate with dual-oxide TFET has been investigated with reduced ambipolar behavior and subthreshold swing [13]. Furthermore, an analytical model of a dual-material single-gate doping-less TFET with gate underlap regions has been proposed [14]. A dual-material gate GaAs/InAs/Ge junctionless TFET has been proposed based on intraband tunneling and interband tunneling with improved SS and I_ON_ [15]. A dual-material gate-oxide-stack double-gate TEFT has also been investigated as a biosensing element, and the underlying device sensitivity has been estimated [16]. However, the effect of dual-material polarity gates on TFET devices has not yet been investigated.

We present the application of a dual-material polarity gate (DMPG) to an in-built N^+^ pocket ED-TFET in this paper. In 2D device simulations, we demonstrate that engineering the dual-polarity gates’ work functions enables the optimization of the ON-current I_ON_, the OFF-current I_OFF_, and the average subthreshold slope and analog/RF performance by simultaneously optimizing the work functions of the dual-polarity gates.

In this work, we investigate the device design and DC and analog/RF performances of the proposed DMPG ED-TFET with regard to several key parameters. A description of the physical structure used in the simulation is presented in Section 2. In Section 3, comparative results and analyses are presented. Finally, Section 4 summarizes the paper.

## 2. Device Structure and Simulation Model

**Figure 1:**
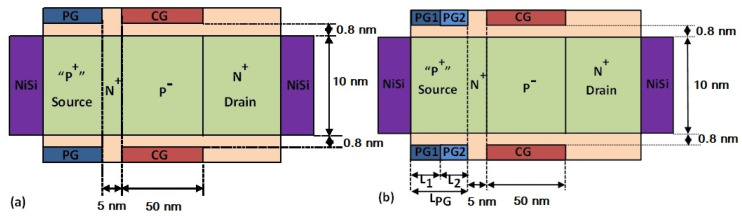
Cross-sectional views of (**a**) the conventional SMPG ED-TFET and (**b**) the proposed DMPG ED-TFET.

**Figure 2:**
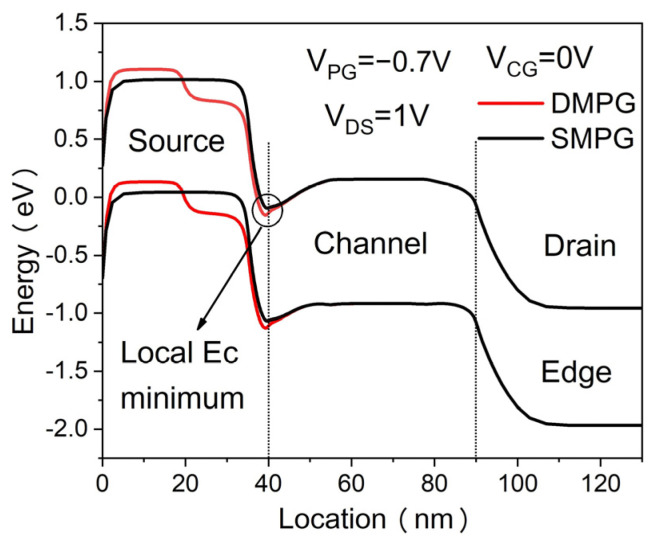
Energy band diagrams of DMPG and SMPG ED-TFET at 1 nm below the Si/oxide interface.

Figure 1a,b illustrate the cross-sectional views of the conventional single-material polarity gate (SMPG) ED-TFET and the proposed dual-material polarity gate (DMPG) ED-TFET. In both types of devices, there are two sets of gate electrodes: control gates (CG) and polarity gates (PG). For comparison, an SMPG ED-TFET with the same channel length is used. The proposed DMPG ED-TFET has two polarity gates with different work functions, denoted by PG1 and PG2, which are set to 4.97 and 4.5 eV, respectively, corresponding to the values of some common metals, as shown in Figure 1b. Initially, L_1_ and L_2_ are set to 20 nm, and the total length of the polarity gate is fixed at 40 nm. In Table 1, we show the detailed design parameters we used in our simulation. During the simulation, the lengths and work functions of the two polarity gates can be varied. As shown in Table 1, both devices have the same doping concentration at the source, channel, and drain, with a starting NPN structure. A PNPN TFET structure is achieved by setting up polarity gates on the source side of the device based on the polarity bias concept. The narrow N^+^ pocket is, therefore, built into the device without the need for additional doping processes, thereby simplifying manufacturing. In general, the proposed DMPG ED-TFETs have the same working mechanism as the conventional SMPG ED-TFETs, except for the dual-material polarity gate configuration. In Figure 2, the energy band diagrams for the proposed DMPG and conventional SMPG ED-TFET at 1 nm below the Si-oxide interface are shown. It appears that the conduction energy band edge (E_C_) at V_CG_ = 0 V has a local minimum point. By aligning the local minimum with the valence energy band edge (E_V_) at the source, the introduction of the N^+^ pocket results in a decrease in the E_C_ curve and a rapid decrease in the tunneling barrier width. However, the incorporation of the DMPG leads to a reduction in the local E_C_ minimum, as shown in Figure 2, and the tunneling barrier width can be further reduced due to the work function modulation of the DMPG.

In this paper, we used the Silvaco Atlas device simulation software (version 5.19.20.R) [17] to perform all the simulations. Based on [18], we validated our simulation model using a non-local band-to-band tunneling (BTBT) model. Based on the analysis of the energy band diagrams, a non-local BTBT model was used to calculate the tunneling probability along the lateral direction of the device. A fine mesh was used across the region of tunneling in the simulations to perform non-local BTBT. Approximating the evanescent wavevector was performed by Atlas using the Wentzel–Kramer–Brillouin method. To include the effect of the electric field on mobility degradation, we used the Lombardi mobility model. In addition, Fermi Dirac and the Shockley–Read–Hall (SRH) recombination models were used. In order to account for the high concentration of doping in the devices, a band-gap narrowing (BGN) model was used. Because the thickness of the silicon film exceeded 7 nm, quantum mechanical effects were not taken into account [19,20].

## 3. Simulation Results and Discussion

The DC and analog/RF characteristics of the proposed DMPG ED-TFET are investigated and compared with those of a corresponding compatible SMPG ED-TFET. The influences of the key parameters are further analyzed in this section.

### 3.1. DC Characteristics

**Figure 3:**
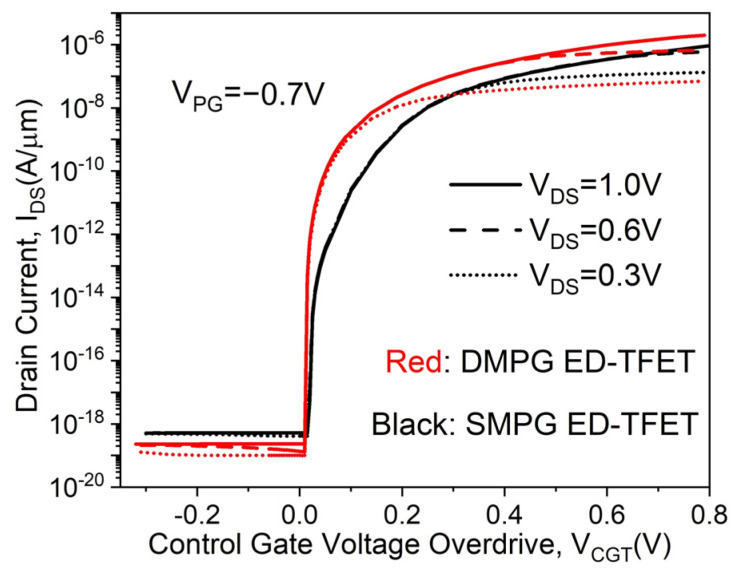
Transfer characteristics of the proposed DMPG ED-TFET and conventional SMPG ED-TFET for various V_DS_.

**Figure 4:**
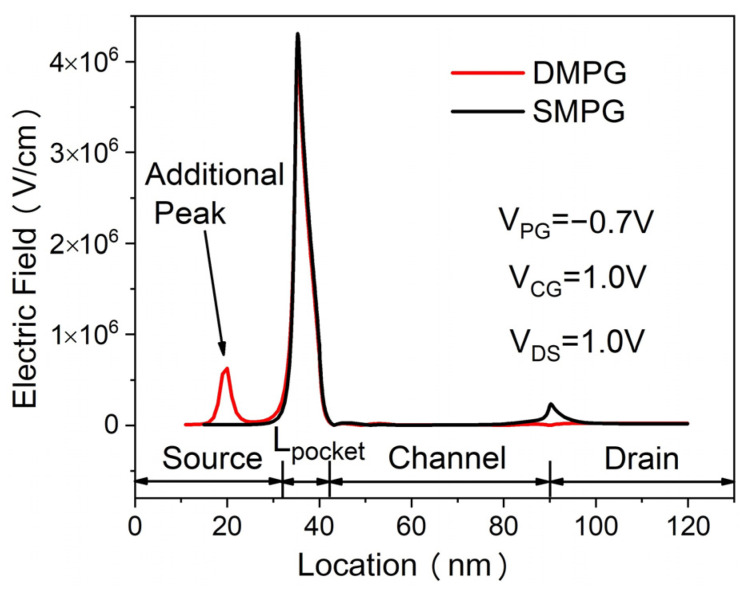
Electric field distributions of DMPG and SMPG ED-TFET at 1 nm below the Si/oxide interface.

**Figure 5:**
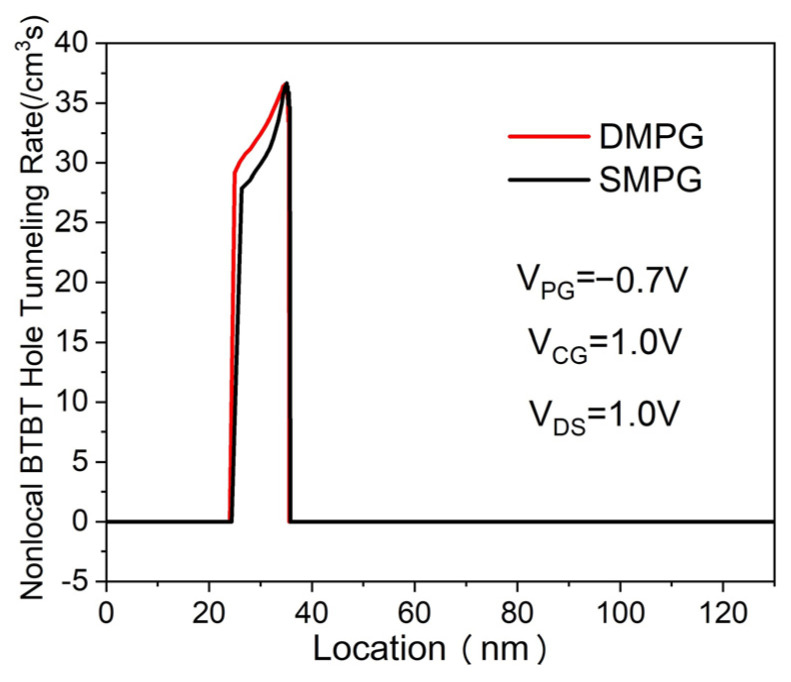
Non-local BTBT hole tunneling rate of DMPG and SMPG ED-TFET at 1 nm below the Si/oxide interface.

The transfer characteristics of the proposed DMPG ED-TFET and conventional SMPG ED-TFET for various drain voltages are shown in Figure 3. The control gate voltage overdrive V_CGT_ is defined as V_CGT_ = V_CG_ − V_TH_, where V_CG_ is the control gate voltage and V_TH_ is the threshold voltage referring to the control gate voltage when the device is turned on. It is clearly seen from Figure 3 that the SS is significantly improved in the proposed device and I_ON_ (calculated at V_CG_ = V_DS_ = 1.0 V, V_PG_ = −0.7 V) is also higher. This can be explained using the electric field distributions, which are shown in Figure 4. By using the dual-material polarity gates of work functions 4.97 eV and 4.5 eV in the proposed device, an additional peak electric field is created in the source region, which provides more acceleration to the tunneling carrier and enhances the non-local BTBT hole tunneling rate near the N^+^ pocket, as shown in Figure 5.

### 3.2. Device Optimizations

**Figure 6:**
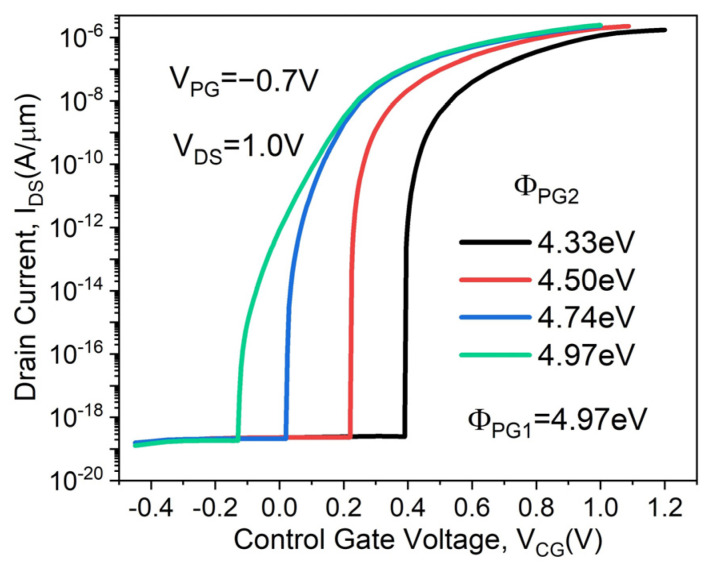
Transfer characteristics of the proposed DMPG ED-TFET for various PG2 work functions.

**Figure 7:**
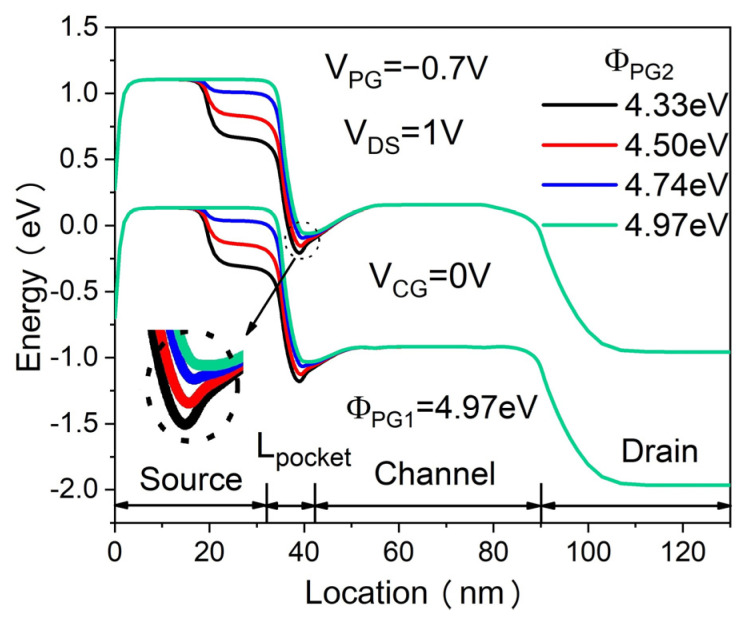
Energy band diagrams of the proposed DMPG ED-TFET for various PG2 work functions at 1 nm below the Si/oxide interface.

The transfer characteristics of the proposed DMPG ED-TFET for various PG2 work functions (Φ_PG2_) are shown in Figure 6. The PG1 work function is fixed at 4.97 eV. The Φ_PG2_ = 4.97 eV corresponds to that of the SMPG ED-TFET. As shown in Figure 6, both I_ON_ and SS increase and the devices turn on at a lower control gate voltage with increasing Φ_PG2_. We show the energy band diagrams of the proposed DMPG ED-TFET for various PG2 work functions at 1 nm below the Si/oxide interface in the OFF-state in Figure 7. For clarity, the band diagram near the local E_C_ minimum is enlarged. From Figure 7, it can be seen that increasing the PG2 work function leads to a reduced depth of the E_C_ well where the local minimum point is located, which results in band-to-band tunneling difficulties. Thus, SS degrades considerably when Φ_PG2_ reaches 4.97 eV. Considering I_ON_ and SS, the optimal work function of PG2 is 4.5 eV when keeping the work function of PG1 fixed at 4.97 eV.

**Figure 8:**
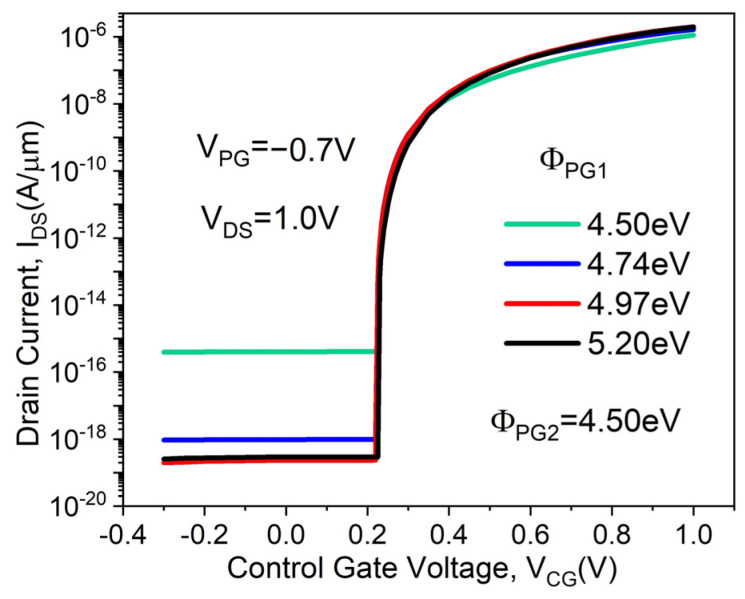
Transfer characteristics of the proposed DMPG ED-TFET for various PG1 work functions.

**Figure 9:**
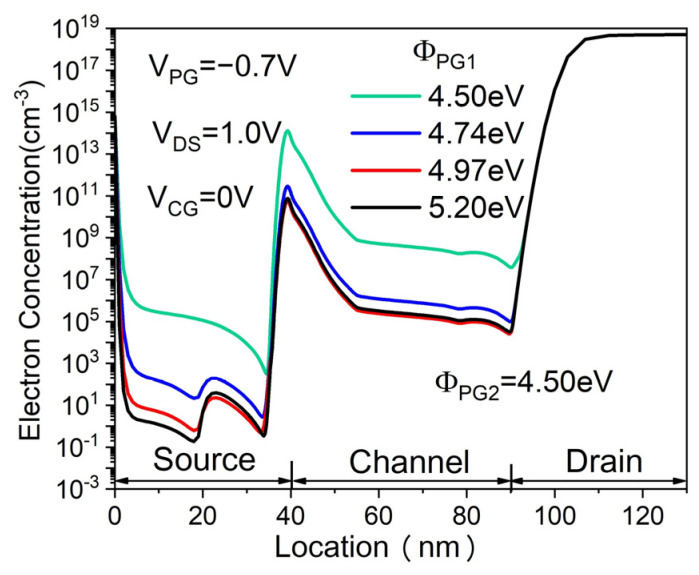
Electron concentration of the proposed DMPG ED-TFET for various PG1 work functions.

Figure 8 shows the transfer characteristics of the proposed DMPG ED-TFET for various PG1 work functions (Φ_PG1_) while keeping Φ_PG2_ fixed at 4.5 eV. The Φ_PG1_ = 4.5 eV corresponds to that of the SMPG ED-TFET. In general, the OFF-state current I_OFF_ (calculated at V_CG_ = 0 V, V_DS_ = 1.0 V, V_PG_ = −0.7 V) decreases with increasing Φ_PG1_, as shown in Figure 8. However, SS, V_TH,_ and I_ON_ are virtually unchanged since Φ_PG2_ is fixed. This can be explained by the fact that in the ON-state, the band-to-band tunneling occurs at the junction between the source region and pocket. Thus, an increase in Φ_PG1_ does not change the band diagram near tunneling significantly. The device with Φ_PG1_ of 4.97 eV has the lowest I_OFF_, as shown in Figure 8. This can be understood from the electron concentration distribution for different PG1 work functions in the OFF-state. In the case of the proposed device with Φ_PG1_ = 4.97 eV, the electron concentration in the channel shows the lowest value, as illustrated in Figure 9. This reduced electron concentration affects the conduction band profile in the OFF-state. As a result, the conduction band well becomes wider, resulting in a lower I_OFF_. When the PG2 work function is fixed at 4.5 eV, the optimal PG1 work function is 4.97 eV.

**Figure 10:**
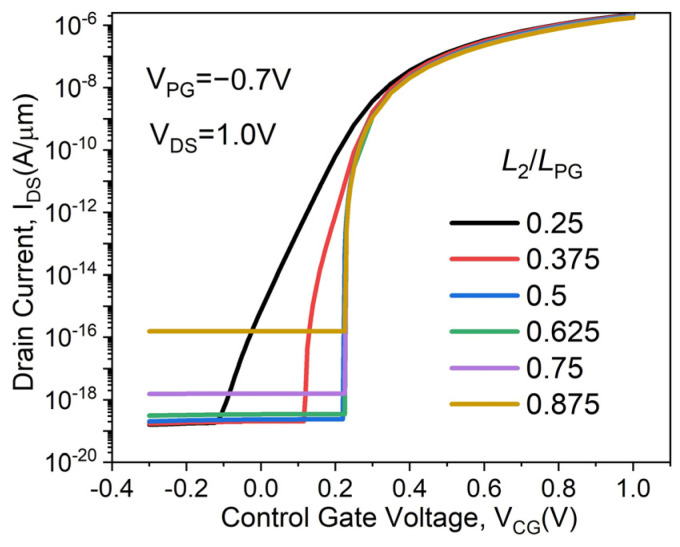
Transfer characteristics of the proposed DMPG ED-TFET for various L_2_/L_PG_ ratios.

**Figure 11:**
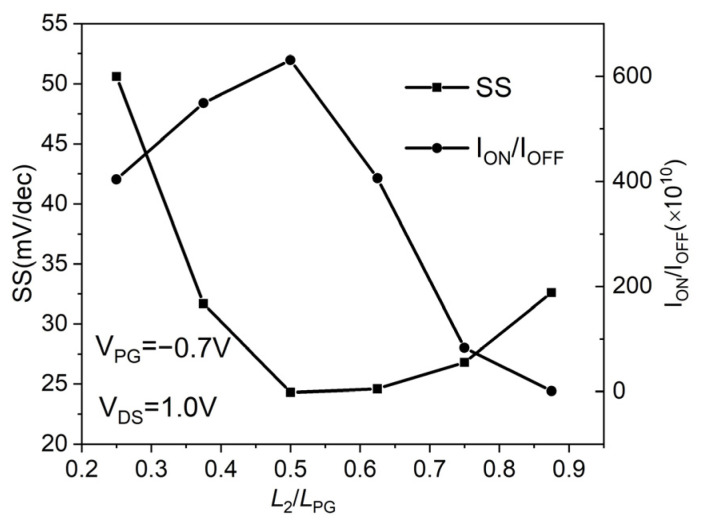
SS and I_ON_/I_OFF_ of the proposed DMPG ED-TFET for various L_2_/L_PG_ ratios.

The transfer characteristics of the proposed DMPG ED-TFET are studied when the L_2_/L_PG_ ratio is varied from 0.25 to 0.875 by changing L_2_ from 10 to 35 nm with a fixed polarity gate length of L_PG_ = L_1_ + L_2_ = 40 nm, as shown in Figure 10. It is evident that SS degradation occurs when the L_2_/L_PG_ ratio is 0.25 and 0.375. The SS is almost consistent when the L_2_/L_PG_ ratio exceeds 0.5; however, as the ratio increases, I_OFF_ increases as well. For all L_2_/L_PG_ ratios, I_ON_ is essentially the same. In Figure 11, the characteristics of the SS and ON/OFF current ratios (I_ON_/I_OFF_) for the DMPG ED-TFET devices are shown. It can be observed that the lowest SS and the highest I_ON_/I_OFF_ occur at L_2_/L_PG_ = 0.5. Considering the SS and I_ON_/I_OFF_, as well as the actual photolithography conditions, L_2_/L_PG_ = 0.5 seems to be a reasonable optimal value for the proposed DMPG ED-TFET.

### 3.3. Analog/RF Performance

**Figure 12:**
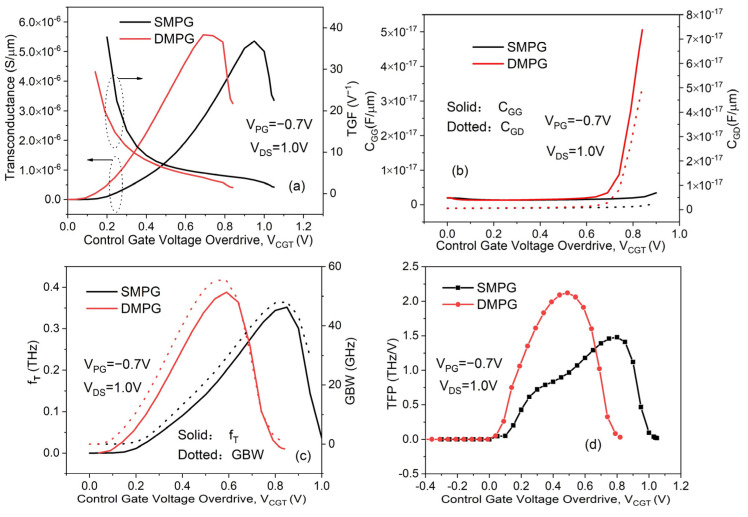
Variation in (**a**) transconductance and TGF, (**b**) C_GG_ and C_GD_, (**c**) *f*_T_ and GBW, and (**d**) TFP versus V_CGT_ of the conventional SMPG and proposed DMPG ED-TFET.

We simulate and compare the analog/RF performance of the proposed DMPG ED-TFETs with that of the conventional SMPG ED-TFETs with identical dimensions, as shown in Figure 12. An analog/RF figure of merit (FoM) consists of the following: transconductance (*Gm*), transconductance generation factor (TGF), gate capacitance (C_GG_), gate–drain capacitance (C_GD_), cutoff frequency (*f*_T_), gain bandwidth product (GBW), and transconductance frequency product (TFP). For fair comparisons, the same Vc_GT_ is used to subtract the effect of the threshold voltage.

This can be expressed as *Gm* = dI_DS_/dV_CG_, where *Gm* is the slope of the log(I_DS_)–V_CG_ curve when V_DS_ remains at 1.0 V. In analog circuits, transconductance is crucial for achieving high gains and *f*_T_. As shown in Figure 12a, the *Gm* of the DMPG ED-TFET is larger than that of the SMPG device. The improved SS of the DMPG structure results in a significant change in I_DS_ with V_CG_, whereas I_ON_ maintains a high value, resulting in a greater *Gm*. Furthermore, *Gm* increases as V_CGT_ is increased until saturation occurs. The maximum *Gm* of the proposed DMPG and conventional SMPG ED-TFETs are 5.57 and 5.35 µS/µm, respectively. Both devices’ transconductance drops rapidly when they enter the saturation region (V_CGT_ of 0.69 V for DMPG, 0.95 V for SMPG). A device’s efficiency can also be quantified by TGF, which represents *Gm* divided by the I_DS_. Figure 12a also shows TGF with varying V_CGT_ for the DMPG and SMPG devices. We can see that the proposed DMPG device has a lower TGF because *Gm* is less dominant than the drain current. Therefore, in spite of the higher *Gm* of DMPG, TGF remains relatively small. As V_CGT_ increases, the drain current increases rapidly, and the TGF decreases accordingly.

As we know, capacitance is an important parameter closely related to the power consumption and switching speed characteristics of transistors. Therefore, variations in C_GG_ and C_GD_ with respect to V_CGT_ of both the proposed DMPG and conventional SMPG ED-TFETs are shown in Figure 12b. It has been observed that C_GG_ and C_GD_ in the proposed DMPG device are higher than those in the conventional SMPG device. When V_CGT_ exceeds 0.6 V, both capacitances of the DMPG device increase rapidly as V_CGT_ increases. For the SMPG devices, the capacitances increase slowly. Other important parameters for RF applications are the cutoff frequency (*f*_T_) and the gain bandwidth product (GBW). At the cutoff frequency, the short-circuit current gain reaches unity and is represented by *f*_T_ = *Gm*/2πC_GG_. For high-frequency circuits, it is generally beneficial to have a high *f*_T_ to ensure that the device can be used widely. The GBW can be expressed as a ratio of *Gm* to C_GD_ for a DC gain value equal to 10, and it is represented by GBW = *Gm*/2π10 C_GD_. It can be inferred from Figure 12c that both *f*_T_ and GBW are improved in the DMPG ED-TFET, which is similar to the trends in Figure 12a. Based on the formulas listed above, the values of *f*_T_ and GBW are both proportional to *Gm*, so the changing trends are also similar. As V_CGT_ further increases, *Gm* drops sharply and the capacitance increases, resulting in a decrease in *f*_T_ and GBW. The proposed DMPG and conventional SMPG ED-TFETs achieve a maximum *f*_T_ of 0.388 and 0.352 THz, and a maximum GBW of 55.52 and 48.01 GHz at V_CGT_ of 0.59 V and 0.85 V, respectively.

Another important FoM for high-frequency circuits is the TFP, which is essentially calculated by multiplying the TGF by the *f*_T_, or TFP = (*Gm*/I_DS_) × *f*_T_. As shown in Figure 12d, the proposed DMPG ED-TFET exhibits higher TFP values than the conventional SMPG ED-TFET due to its higher *f*_T_. The DMPG and SMPG devices achieve a maximum TFP of 2.12 and 1.48 THz/V at V_CGT_ of 0.49 V and 0.8 V, respectively. Compared to conventional SMPG ED-TFETs for low-voltage circuits, the proposed DMPG ED-TFETs appear to be more suitable for RF applications.

## 4. Conclusions

In this paper, we presented a device structure that incorporates a dual-material gate in a PNPN ED-TFET based on the polarity bias concept. The dual-material gate was used on the polarity gate, which was biased at −0.7 V to induce a P^+^ region in the source. By introducing an additional electric field peak, we demonstrated that the DMPG architecture further improves the drive current and SS characteristics. Furthermore, the device design was optimized by modulating the work functions of PG1 and PG2 and the L_2_/L_PG_ ratio. In general, L_2_/L_PG_ = 0.5, PG1 work function Φ_PG1_ = 4.97 eV, and PG2 work function Φ_PG2_ = 4.5 eV are recommended for DMPG ED-TFET. Two-dimensional simulations were used to evaluate DC and analog/RF performance. The simulated performance of the DMPG ED-TFET performed better than that of the conventional SMPG ED-TFET at the optimized dimensions of SS, I_ON_, *Gm*, TGF, *f*_T_, GBW, and TFP in low-voltage situations. Based on this, we anticipate that the circuit performance would be better with the DMPG architecture.

## Figures and Tables

**Table 1:** Parameters used for device simulation.

Parameter	Conventional SMPG ED-TFET [3]	Proposed DMPG ED-TFET
Source Doping	4 × 10^19^ cm^−3^ (N^+^)	4 × 10^19^ cm^−3^ (N^+^)
Channel Doping	1 × 10^17^ cm^−3^ (P^−^)	1 × 10^17^ cm^−3^ (P^−^)
Drain Doping	5 × 10^18^ cm^−3^ (N^+^)	5 × 10^18^ cm^−3^ (N^+^)
CG Work function	4.74 eV	4.74 eV
PG Work function	4.33 eV	-
PG1 Work function	-	4.97 eV
PG2 Work function	-	4.5 eV

## Data Availability

The data presented in this study are available on request from the corresponding author. The data are not publicly available due to privacy.
